# Helium ion microscopy of enamel crystallites and extracellular tooth enamel matrix

**DOI:** 10.3389/fphys.2014.00395

**Published:** 2014-10-10

**Authors:** Felicitas B. Bidlack, Chuong Huynh, Jeffrey Marshman, Bernhard Goetze

**Affiliations:** ^1^Department of Mineralized Tissue Biology, Forsyth InstituteCambridge, MA, USA; ^2^Department of Developmental Biology, Harvard School of Dental MedicineBoston, MA, USA; ^3^Carl Zeiss Microscopy LLC, One Corporation WayPeabody, MA, USA

**Keywords:** tooth enamel, matrix organization, amelogenin, immuno-gold labeling, helium ion microscopy, high-resolution microscopy

## Abstract

An unresolved problem in tooth enamel studies has been to analyze simultaneously and with sufficient spatial resolution both mineral and organic phases in their three dimensional (3D) organization in a given specimen. This study aims to address this need using high-resolution imaging to analyze the 3D structural organization of the enamel matrix, especially amelogenin, in relation to forming enamel crystals. Chemically fixed hemi-mandibles from wild type mice were embedded in LR White acrylic resin, polished and briefly etched to expose the organic matrix in developing tooth enamel. Full-length amelogenin was labeled with specific antibodies and 10 nm immuno-gold. This allowed us to use and compare two different high-resolution imaging techniques for the analysis of uncoated samples. Helium ion microscopy (HIM) was applied to study the spatial organization of organic and mineral structures, while field emission scanning electron microscopy (FE-SEM) in various modes, including backscattered electron detection, allowed us to discern the gold-labeled proteins. Wild type enamel in late secretory to early maturation stage reveals adjacent to ameloblasts a lengthwise parallel alignment of the enamel matrix proteins, including full-length amelogenin proteins, which then transitions into a more heterogeneous appearance with increasing distance from the mineralization front. The matrix adjacent to crystal bundles forms a smooth and lacey sheath, whereas between enamel prisms it is organized into spherical components that are interspersed with rod-shaped protein. These findings highlight first, that the heterogeneous organization of the enamel matrix can be visualized in mineralized en bloc samples. Second, our results illustrate that the combination of these techniques is a powerful approach to elucidate the 3D structural organization of organic matrix molecules in mineralizing tissue in nanometer resolution.

## Introduction

A major hurdle for our understating of tooth enamel formation has been to analyze simultaneously protein and mineral phase in developing teeth. This is because tooth enamel is a composite material consisting of proteins and calcium phosphate crystallites that are extremely small and needle shaped. With a thickness that is only a few nanometers (Daculsi et al., 1984) the crystallites are far below the resolution limit of light microscopy, the technique of choice for conventional histology and immunohistochemistry analyses. However, the increasing mineral content in later stages of enamel development prevents the thin sectioning and the use of these classical histology methods to visualize the proteins in the un-demineralized specimen. The specimen has to be demineralized to allow for the preparation of thin sections followed by labeling or staining, and analysis using light microscopy. Electron microscopy is classically the technique of choice to study the formation and arrangement of the mineral phase. In early stages of enamel formation the mineral content of the enamel is still low enough to allow for ultra-thin sectioning and labeling with immuno-gold, if desired, and the use of transmission electron microscopy (TEM) analyses, including selected area electron diffraction to identify the mineral phase. Using this approach, it has been shown that enamel mineral formation starts as amorphous calcium phosphate (ACP) (Beniash et al., 2009). Thus, the protein matrix guides ACP maturation to form the correct shape of hydroxyapatite-like (HAP) crystallites (Moradian-Oldak, 2012). At later stages of enamel formation, the higher mineral content prevents the preparation of ultra-thin sections for TEM analyses. Scanning electron microscopy (SEM) is then frequently used to study the appearance of tooth surfaces, or to analyze polished and etched sections of the tooth to learn about the thickness and degree of mineralization of the enamel layer, as well as its microstructure. SEM analyses of fractured tooth surfaces give a spatial impression of enamel microstructure without prior etching.

Of the three structural tooth enamel matrix proteins amelogenin, ameloblastin, and enamelin, it is amelogenin that constitutes, with about 90 weight percent, the bulk of the organic matrix (Fincham et al., 1999). Although amelogenin is required for proper enamel formation, it is reabsorbed into the secreting ameloblast cells. The organic enamel matrix is therefore ephemeral and changes in composition and structural organization dynamically throughout enamel formation.

Since the formation and arrangement of enamel crystallites is protein-guided, it is desirable to analyze simultaneously in a given specimen both mineral and organic phases to better understand their interactions and spatial organization. One method with sufficient analytical resolution to visualize both the small crystallites and specifically labeled proteins is the use of TEM for the detection of immunogold-labeled proteins in ultra-thin sections of mineralized tissue. However, this technique cannot visualize the three dimensional structural organization of protein and mineral phase (Nanci et al., 1994; Diekwisch et al., 1995). Other high-resolution techniques such as atomic force microscopy, freeze fracture techniques, or cryo-TEM have delivered valuable new insights (Robinson et al., 1981; Beniash et al., 2005; He et al., 2008) but do not allow for the distinction between different matrix proteins in the organic phase. This poses a critical problem because the highly ordered composite protein-mineral structure results from key interactions between matrix proteins, growing crystals, and changes in the mineralizing matrix. These protein-protein and protein-mineral interactions regulate the final morphology and arrangement of hydroxyapatite-like crystals and, as a consequence, the mechanical properties of the resulting mature mineralized tissue (Margolis et al., 2006; Beniash, 2011; Moradian-Oldak, 2012).

This study pioneers the use of a new imaging technique, helium ion microscopy (HIM), to study enamel and addresses the need to analyze simultaneously the spatial organization of matrix proteins and crystallites *in situ* to advance our understanding of tooth enamel formation (Hill et al., 2012; Notte and Goetze, 2014). HIM has, as a novel imaging technique, recently received a lot of attention because it allows for imaging at nanometer resolution with outstanding depth of field. HIM enables us to look in a new way at a wide array of sample preparations since there are no constraints, as in TEM or light microscopy, on sample concentrations, thickness of the sample, or the opacity of the substrate. In many respects, the sample preparation for HIM and SEM are very similar. However, as discussed below, there are important differences between these two techniques such as the mechanisms of contrast generation and image formation. So far, HIM has mostly been applied in material sciences. This paper presents a comparison between FE-SEM and HIM imaging of developing tooth enamel and introduces HIM as a method of in situ analyses of biomineralization samples. Specifically, we used this high resolution imaging approach to test the hypothesis that the 3D structural organization of the enamel matrix is different in direct vicinity to bundles of forming crystallites compared to the matrix filled space between these crystallite bundles. The identification of differences in matrix organization lays the groundwork for further studies to identify the distribution of the structural proteins of the enamel matrix and their cleavage products.

## Materials and methods

### Sample preparation

All procedures for obtaining the samples followed the Forsyth IACUC approved protocol. Mouse wild type (C57BL/6) hemi-mandibles and hemi-maxillae were fixed in 4% zinc formalin for 24 h at room temperature, rinsed in water and dehydrated through a graded ethanol series followed by gradual ethanol substitution with LR White acrylic resin (Electron Microscopy Sciences, Hatfield, PA). The samples were polymerized at 60°C for 24 h and, after cooling to room temperature, polished in a parasagittal plane to expose the area of interest within the tooth enamel. Etching of the polished surface for 15 s in 0.1 M phosphoric acid (Sigma Aldrich) exposed the enamel matrix between and around forming enamel crystal bundles. After air-drying, samples were mounted on electron microscopy stubs and viewed in HIM and FE-SEM.

Tooth enamel at various stages of development was used for the imaging, starting with early secretory enamel, including late secretory, transition stage, and maturation stage enamel. These different stages of enamel development are very well defined and characterized (Smith et al., 2011) in relation to external landmarks and distance measurements from the cervical loop. These reference points were adapted from the work of Smith and Nanci (1989), as adapted by Lacruz et al. (2012) and are visible also in embedded samples. This procedure ensured that precisely defined and comparable enamel regions and mineralization stages were analyzed.

#### Immunohistochemistry

Commercially available primary antibodies were used to label the C-terminus of the full-length amelogenin molecule (Abcam ab59705). The primary antibody for the full-length amelogenin was applied at a 1:1000 dilution and then identified using a goat anti-rabbit IgG conjugated to 10 nm immuno-gold secondary antibody labeling (Aurion, obtained through Electron Microscopy Sciences, Hatfield, PA, USA). Specifically, the embedded, polished and etched samples were transferred into phosphate buffered saline (1× PBS), rinsed twice for 2 min, then treated with 2% sodiumborohydrate solution for a total of 90 min at room temperature with four solution changes, followed by five rinses in 1× PBS of 2 min each and 20 min in 4% goat serum at RT. The primary antibody was applied in 4% goat serum overnight at 4°C. The sample was rinsed twice for 5 min in 1× PBS before the secondary antibody was applied at 1:25 dilution for 2 h at RT followed by 4 h at 4°C. The sample was then washed five times for 5 min in 1× PBS, followed by five 2-min rinses in distilled water and air-dried. Negative controls were processed the same way, except that the addition of primary antibody was omitted during incubation with 4% goat serum overnight at 4°C. It has been shown previously that the goat anti-rabbit IgG conjugated immunogold label does not bind unspecifically to mineral or enamel matrix protein (Du et al., 2009).

### Imaging

All samples were imaged without any coating. SEM was performed on a Zeiss Merlin FE-SEM at Zeiss LLC., headquarters in Peabody, MA, and a Zeiss Ultra Plus FE-SEM at the Harvard Center for Nanoscale Structures. With both instruments, samples were viewed at a working distance of 3–4 mm, a voltage of 1 kV, and 50–52 pA probe current.

For HIM either the Zeiss Orion® helium ion microscope located at MIT or the instrument at the Zeiss headquarters in Peabody was used and samples were viewed at 9–11 mm working distance and flood gun settings optimized for each sample.

**Figure 1:**
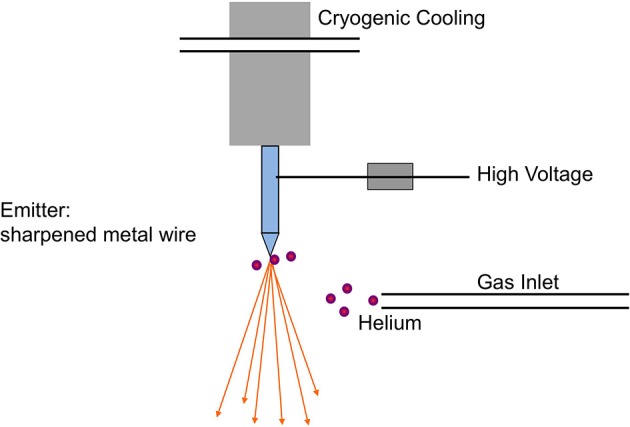
**Schematic of the field emission source in the helium ion microscope**. The crystalline metal wire tip, chemically etched to form a very pointy tip, is cooled to about 80 K and located in a high vacuum chamber, which is filled with helium gas during operation. The helium atoms are ionized in the high electric field at the end of the wire tip and accelerated to form the helium ion beam with an appropriate voltage between the emitting tip and an extractor. Illustration simplified from Hill et al. (2012).

**Figure 2:**
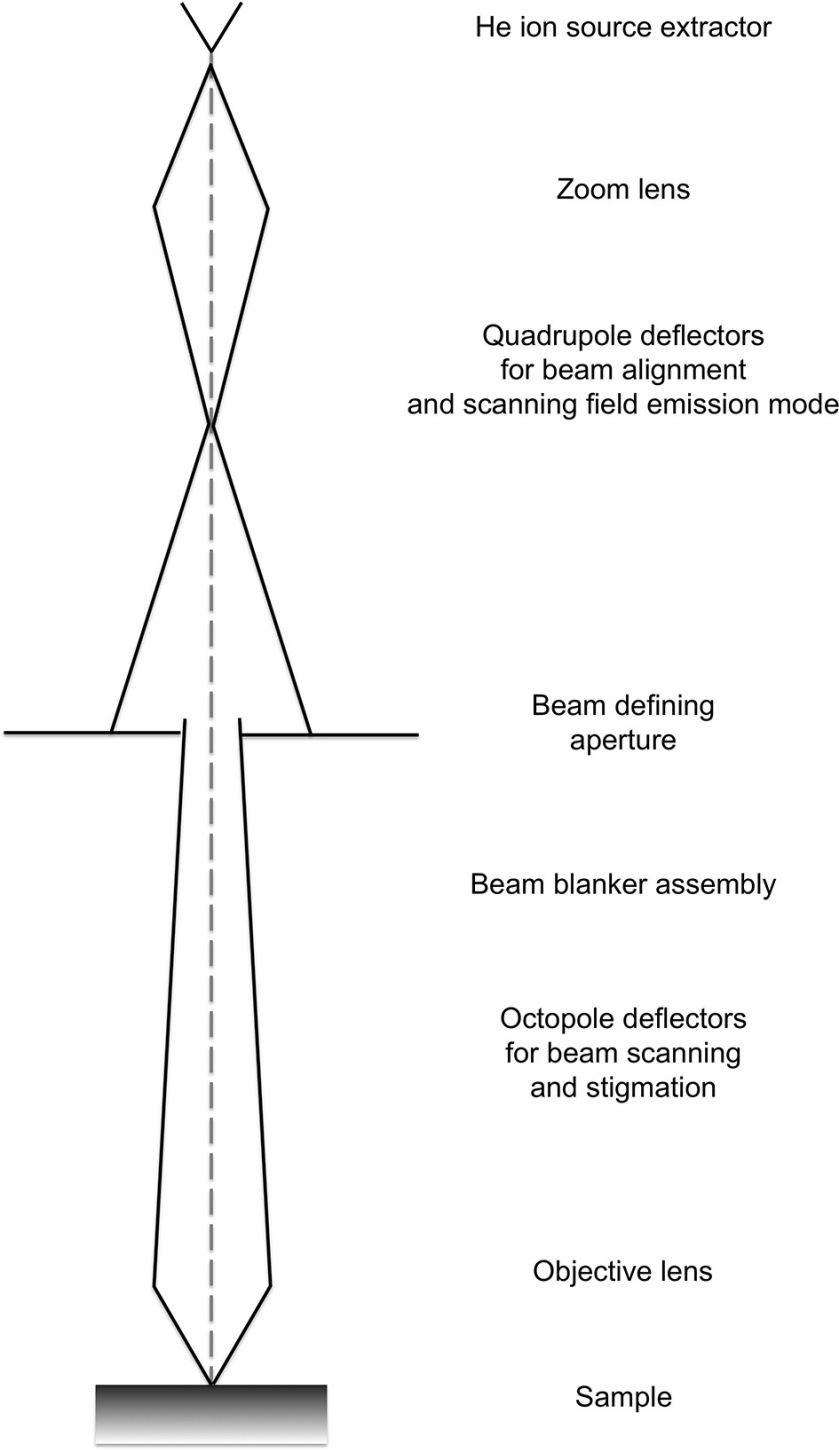
**Schematic showing the typical beam envelope and principal components of a helium ion microscope column**. Illustration modified from the comprehensive description of helium ion microscopy (Hill et al., 2012).

The ion beam for imaging is generated in the HIM by introducing helium ions to a cryogenically cooled tungsten tip, a crystalline metal wire that is chemically etched to form a very pointy tip, the shape of a pyramid (Figure 1). The shape of the tip is refined through field evaporation of atoms from the tip, which results in atomically sharp edges and only a few atoms protruding from the tip. For operation, only three atoms forming a trimer are used as emitting source and this process is thus referred to as building the trimer. When properly aligned, the helium ions interact with the emitting tungsten tip such that only the emission from one atom, seen as the brightest one of the trimer, is used for imaging. Changing the gas pressure modifies the beam current. The beam path and the shape of the beam envelope are schematically illustrated in Figure 2. The deBroglie wavelength of the accelerated helium ions is about 100 times smaller than the corresponding voltage dependent wavelength of electrons used in SEM. Compared to the Schottky FEG electron source in field emission scanning electron microscopes (FE-SEM), the source brightness in HIM is about 30 times higher (Bell, 2009). These factors, and the small energy spread of the HIM source, contribute to the superb sub-nanometer spatial resolution and great contrast in HIM images of uncoated specimens and provide different information than SEM. In comparisons between HIM and SEM imaging, it has been shown that the interactions of helium ions with the sample material during imaging are confined to a very small volume at the sample surface (Bell, 2009). This beam-sample interaction in the HIM is similar to the effect of SEM imaging at very low voltage.

**Figure 3:**
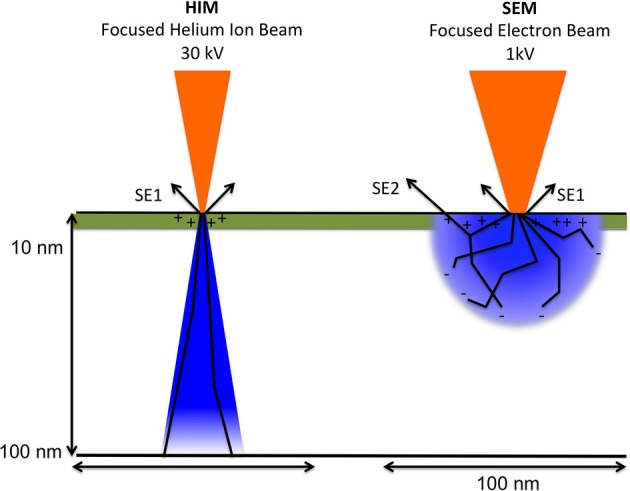
**Schematic comparing between helium ion beam and electron beam and charge distribution upon beam-sample interaction**. The volume of sample interaction, depicted in blue, represents the volume affected by the incident ion or electron beam. It depends on the atomic composition of the sample (Z number), as well as the energy and angle of the incident beam. Arrows labeled SE1indicate secondary electrons created by the primary beam. SE2: secondary electrons created from back scattered electrons. The escape depth of secondary electrons is indicated in green. Illustration based on the comprehensive description of helium ion microscopy (Hill et al., 2012) and the most recent description of advances in HIM imaging (Notte and Goetze, 2014).

However, different from SEM imaging, and illustrated in Figure 3, only a positive net charge is created by the helium ion beam at the very top surface layer because they readily obtain electrons and thus become neutralized when passing into bulk matter. A low-energy electron flood beam in the Orion HIM neutralizes this positive charge. Both energy as well as duration of the flooding with the neutralizing beam can be adjusted and performed either after each line or after each frame when the sample is scanned. During imaging with the helium beam, the flood gun is blanked, while during neutralizing of charges the helium beam is blanked and the secondary electron detector is biased negatively, thus preventing saturation. This imaging approach allows for the analysis of insolating samples, such as biological samples, at nanometer resolution and without application of conductive coating. Furthermore, the helium beam has, compared to the electron beam, very little angular deflection upon entering the sample due to the high mass of helium ions compared to electrons. Since electrons are much lighter they experience much higher deflection angles after entering the bulk sample.

The surface sensitivity of HIM also mandates that the surface of a given sample be free of contamination with hydrocarbons that can result from sample preparation steps such as a final rinse of the sample with methanol to evaporate water, or from using canned air often used in SEM sample preparation to blow off the sample surface. Also, if a given sample is to be analyzed by different techniques in a workflow, HIM analyses should precede SEM analyses because the interactions between electron beam and sample in the SEM can result in beam damage and surface alteration, especially when uncoated organic samples are viewed. As a consequence, the generation of hydrocarbons on the sample surface generates a layer of atoms that interact with the helium beam. The sample appears as if out of focus, or as if covered by a blanket, it could be described as the “table cloth effect.”

Despite apparent similarity in sample preparation between SEM and HIM, such as mounting the sample on the same metal stubs and viewing desiccated samples under high vacuum, the differences between these two techniques provide different images of the same sample and different kinds of information.

## Results

### Helium ion microscopy

HIM imaging is extremely sensitive to contamination of the sample surface with hydrocarbons, which result in loss of contrast and depth of field. This surface and contamination sensitivity requires that methods of sample preparation be optimized for HIM. For example, a final rinse of the sample in methanol to accelerate water evaporation after a final washing step, or the use of canned air to blow a sample dry, can result in a layer of hydrocarbons resembling a table cloth on the surface that prevent the detection of any surface details, but no such effect is seen in SEM analyses. Protocols for sample preparation and storage have been optimized for HIM to ensure clean and contamination free sample surfaces. Samples viewed in SEM and HIM for comparison were always prepared and stored exactly the same way.

**Figure 4:**
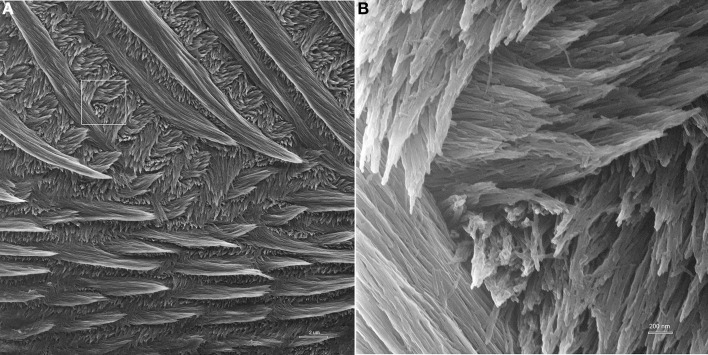
**Helium ion micrograph of maturation stage mouse incisor enamel, polished and etched in parasagittal plane**. The sample is uncoated. **(A)** View over enamel prism organization close to the labial tooth surface. The excellent contrast and resolution permit, despite low magnification, the discernment of enamel crystallites within the prisms. Scale bar 2 μm. The area indicated in A is magnified in **(B)** and illustrates the excellent resolution and depth of field of helium ion microscopy at higher magnification where single crystallites can clearly be distinguished.

As seen in Figure 4, HIM provides exceptional depth of field and resolution. The low magnification imaging of mature mouse incisor enamel shows that the technique provides good contrast and allows for the discernment of crystallites within enamel prisms at low magnifications and in overview images with a field of view of 30 μm (Figure 4A). At high magnification, with field of view of 3 μm, single enamel crystallites are crisp and clearly seen in all of their different orientations and throughout the depth of field (Figure 4B).

**Figure 5:**
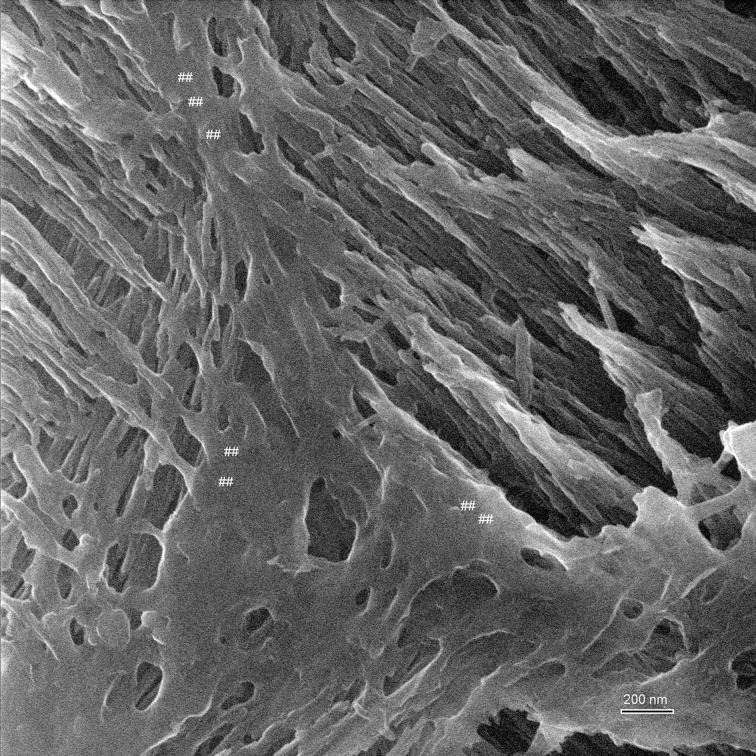
**Helium ion micrograph of an uncoated sample of mouse incisor enamel in early maturation stage, polished and etched in parasagittal plane**. The imaged area shows enamel crystallites extending in their long axis in different directions and planes as they belong to different enamel prisms. The great depth of field reveals on the left side of the image crystallites in the foreground extending between upper left to lower right. At a further depth but imaged at equal resolution and contrast intensity, crystallites extend between lower left and upper right. Between the prisms appears smooth organic material (marked ##). Scale bar 200 nm.

Imaging of early maturation stage and transition stage enamel provides a good opportunity to test whether organic matter provides enough contrast and can be discerned from calcium phosphate crystallites. Figure 5 illustrates this point and shows remnants of the organic matrix between enamel prisms. Both mineral and organic matter are seen with very good depth of field, the organic material appears smooth and adjacent crystallites seem embedded in it.

**Figure 6:**
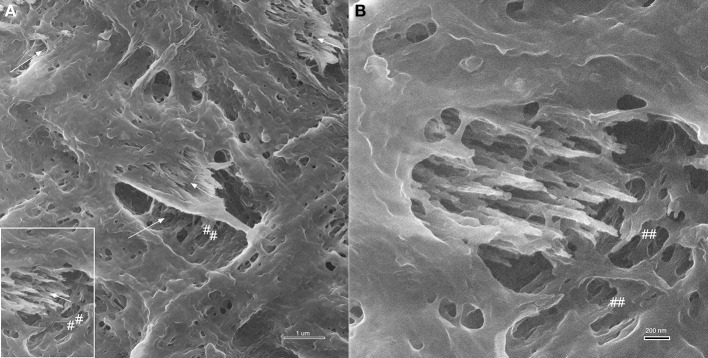
**Helium ion micrographs of mouse incisor enamel in late secretory stage with crystallites (arrows) embedded in organic matrix**. **(A)** Overview image, 9 μm field of view. Crystallites are in bundles and arranged in typical rodent decussation pattern (arrows). Organic material directly adjacent to the prism perimeter has a lacey appearance (marked as ##). Area indicated in **(A)** imaged at higher magnification in **(B)** with a field of view of 3 μm to illustrate the different appearance of the matrix in direct vicinity to crystallite bundles compared to the organic matrix between prisms.

Examples for HIM imaging of late secretory stage enamel are shown in Figure 6. Figure 6A gives an overview at 9 μm field of view and shows bundles of crystallites embedded in the organic matrix (arrows). The typical rodent decussation pattern, with single layers of prisms being oriented perpendicular to each other is visible. The organic material directly adjacent to the prism perimeter has a lacey appearance (marked ##). At higher magnification, a field of view of 3 μm, shown in Figure 6B illustrates the different appearance of the matrix in direct vicinity to crystallite bundles compared to the organic matrix between prisms.

To identify the organic material, we have also analyzed samples with 10 nm immuno-gold labeled amelogenin. However, because immuno-gold particles are covered with protein in the form of the secondary antibody, they have the same contrast as the protein matrix surrounding them. So, using the primary detector in HIM, the immuno-gold label was not detected. That this was not due to the absence of labeling signal was verified by analyzing the same sample and sample area in back scattered electron mode in FE-SEM, where the immuno-gold label can be clearly seen, as described below. Therefore, a backscattered electron detector is required in the HIM to detect this kind of signal. At the time of these analyses, however, the instruments available did not have this set up.

### Field emission scanning electron microscopy (FE-SEM)

**Figure 7:**
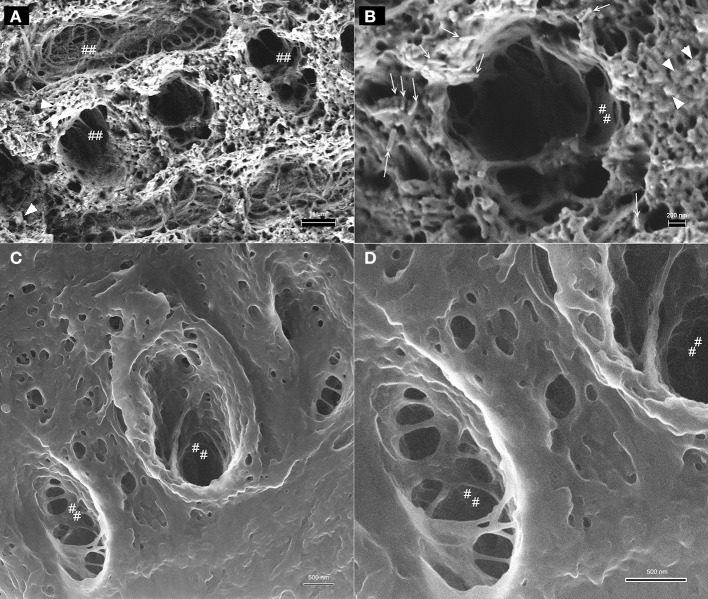
**Comparison between imaging in FE-SEM and HIM imaging of organic matrix in secretory stage mouse enamel**. The mineral phase of forming prisms has been removed by etching thus exposing the organic matrix. **(A,B)** FE-SEM images show the variable structure of the organic matrix, the spaces accommodating the forming prisms appear as a black hole, however, due to limited depth of field. Organic matrix forming the wall, as it were, of the prism spaces is smooth and similar to lace as indicated (marked as ##). Spherical structures of up to ~100 nm diameter are abundant between forming prisms (arrow heads) and rod like features of several hundred nanometer length and less than 100 nm diameter can be discerned (open arrows). **(C,D):** HIM images reveal with great depth of field the network-like organization of organics delimiting the prism space. The matrix between prisms appears smooth and no structural details can be clearly discerned.

A comparison between HIM and FE-SEM analyses of secretory stage enamel is shown in Figure 7 and highlights the differences between these two imaging techniques: the much greater depth of field in HIM imaging combined with helium ion beam-sample interactions that remain very much on the sample surface, on the one hand, and the deeper reaching electron beam-sample interactions in FE-SEM, on the other hand. As a result, organic material in the same stage of enamel development and in equally prepared samples has a different appearance, and provides different information when analyzed in HIM compared to FE-SEM. Figures 7A,B reveal in FE-SEM analysis structural details of the organic matrix surface that are not discernible in HIM (Figures 7C,D). Figures 7B,D show details of Figures 7A,C in higher magnification. The mineral phase of forming prisms has been removed in the etching process thus exposing the organic matrix surrounding the forming prism. Although the HIM images reveal the network-like organization of organics delimiting the prism space (Figures 7C,D), the matrix between prisms appears smooth and no structural details can be clearly discerned. FE-SEM, in contrast, has much less depth of field and the space accommodating the forming prisms appears as a black hole. Yet, the structure of the organic matrix is much clearer and details can be discerned. For example, just as in HIM, the organic matrix forming the wall, as it were, of the prism spaces is smooth and similar to lace as indicated (marked as ##). Interestingly, the matrix between the forming prisms has a completely different organization and a variety of shapes and structures can be seen. Abundant spherical structures of up to about 100 nm diameter can be discerned (Figure 7A arrow heads), as well as rod like features of several hundred nanometer length and less than 100 nm diameter are visible (Figure 7B open arrows).

**Figure 8:**
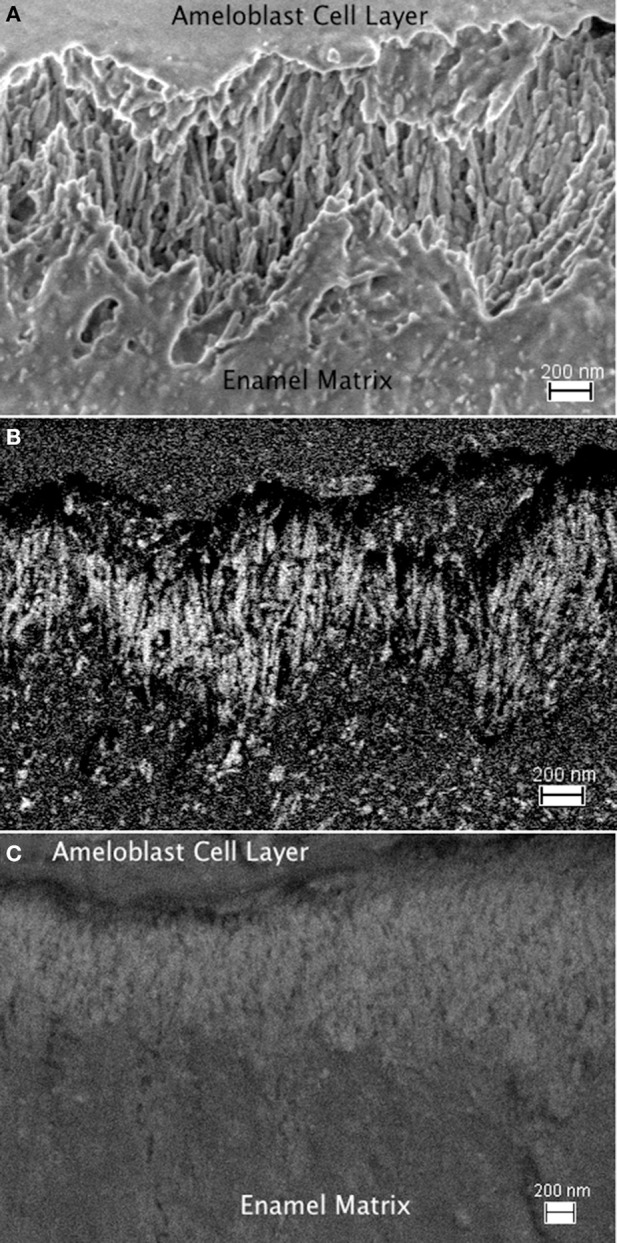
**FE-SEM imagines of enamel matrix organization upon secretion by secretory stage ameloblasts. (A):** The parallel alignment of elongated, rod shaped structures is seen using the in-lens detector. The interface between ameloblast cell layer and secreted matrix is in the top part of the image, the mineralizing matrix is in the bottom part. The time since secretion increases with the distance from the ameloblasts. **(B)** Back scattered electron signal of the same sample area to identify the immuno-gold labeled full-length amelogenin. The electron density of the gold particles results in their bright white appearance. A zig-zag shaped line divides the area of parallel aligned matrix structure from an underlying area of different organization in the lower part of the image with earlier secreted enamel matrix. This zig-zag line follows the shape of the Tomes process of the ameloblasts, which extend from the upper part into the imaged area. **(C)** Control sample imaged in back scattered electron mode. The absence of gold labeling results in a lack of high contrast due to absence of a backscattered signal from gold particles. This implies that in the absence of the primary antibody labeling the C-terminal end of the amelogenin, the immuno-gold particles do not bind nonspecifically, or preferentially to mineral or enamel matrix proteins.

**Figure 9:**
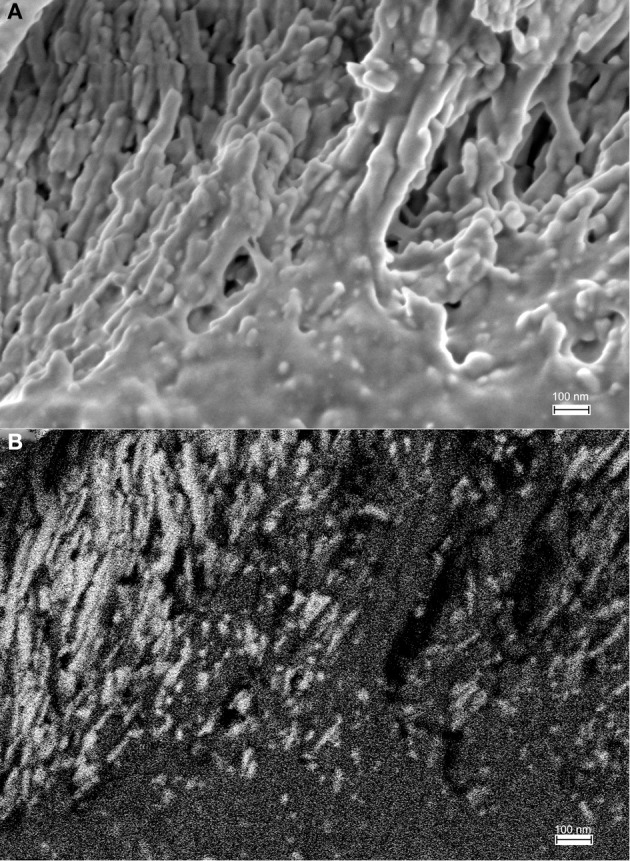
**Rod-shaped structures at the mineralization front, between the ameloblast cell layer (on top of the image area) and the secretory stage enamel matrix is shown in high magnification. (A)** Field of view 1.816 μm imaged using the in-lens detector, 10 nm sized particles can be discerned. **(B)** Backscattered electron image of the same sample area showing the immuno-gold labeled amelogenin in bright white due to the high electron density of gold particles.

Using immunogold labeling of the C-terminal end of amelogenin, the full-length amelogenin or the cleaved C-terminus can be identified, and the enamel matrix organization upon secretion by secretory stage ameloblasts is visible in FE-SEM (Figure 8). The parallel alignment of elongated, rod shaped structures is seen using the in-lens detector (Figure 8A) and identified by the backscattered electron signal of the immuno-gold label as seen in Figure 8B, with the control shown in Figure 8C. The parallel alignment of the labeled protein aggregates is apparent and seems to sit on a zig-zag shaped line that divides the area of parallel rods from an underlying area of different organization. This zig-zag line follows the shape of the Tomes' process of the ameloblasts. The arrangement of rod-shaped structures at the mineralization front, between the ameloblast cell layer (on top of the image area) and the secretory stage enamel matrix is shown in high magnification in Figure 9, where 10 nm sized particles can be discerned using the in-lens detector (Figure 9A) and the backscattered electron detector (Figure 9B).

## Discussion

In the study of tooth enamel formation there exists a divide between *in vitro* studies and the use of animal models and *in situ* analyses of enamel formation. This is partly due to the technical challenges of analyzing the formation of very small calcium phosphate crystals in an organic matrix. This study is an attempt to address this problem by using high-resolution imaging techniques such as HIM and FE-SEM to investigate the relationship between calcium phosphate crystallites and structural organization of the organic matrix *in situ* and in various stages of enamel development.

The most abundant of the three structural enamel matrix proteins, amelogenin, is required for proper enamel formation, but is reabsorbed into the secreting ameloblast cells (Bartlett, 2013). The organic enamel matrix is therefore ephemeral and changes dynamically throughout enamel formation (Simmer et al., 2012). Amelogenin has only one phosphorylation at Ser16 and no other post-translational modifications (Takagi et al., 1984; Fincham and Moradian-Oldak, 1995). Because it has only one posttranslational modification, amelogenin has been successfully expressed in bacteria and, due to its high abundance, can be purified from immature pig teeth. These advantages have afforded *in vitro* studies with sufficient quantities of purified protein, which sets amelogenin apart from the other two enamel matrix proteins ameloblastin and enamelin. A wealth of *in vitro* studies has focused on the self-assembly behavior of amelogenin under a range of conditions, recently reviewed by Moradian-Oldak (2012). Amelogenin forms a gel-like self-assembly structure under physiological conditions of pH, temperature, and concentration (Mechanic et al., 1967; Katz et al., 1969; Wiedemann-Bidlack et al., 2007). Therefore, amelogenin is most frequently studied *in vitro* under non-physiological conditions where nanospheres are observed that, under certain conditions further assemble to form elongated structures (Du et al., 2005; Bromley et al., 2011; Chen et al., 2011; Wiedemann-Bidlack et al., 2011). However, recent evidence indicates that the often described amelogenin nanospheres are not stable in the presence of calcium and/or phosphate ions or when mineralization is initiated (Tarasevich et al., 2009a).

New insights from *in vitro* studies about the interaction of amelogenin with mineral and calcium phosphate crystal surfaces and previous models of enamel formation have provided a basis for expanded, or updated models of enamel mineralization and the role of amelogenin (Robinson et al., 2003; Robinson, 2007; Tarasevich et al., 2009b; Beniash et al., 2012; Moradian-Oldak, 2012). Recent models propose that amelogenin nanospheres (Du et al., 2005), dimers (Martinez-Avila et al., 2012), or barrel structures (Fang et al., 2013) aggregate into linear arrays, comparable to strings of pearls, and form a template that induces apatite formation. The role of inducing apatite formation and guiding crystal arrangement has been suggested for amelogenin by various authors, and has been modified to propose that native full-length amelogenin stabilizes ACP phases, whereas upon cleavage this stabilizing quality is lost and the amorphous mineral transforms into crystalline hydroxyl apatite (Beniash et al., 2009; Yang et al., 2010; Kwak et al., 2011; Moradian-Oldak, 2012).

Combining what is known about the dynamic nature of the enamel matrix *in situ* and *in vitro* findings of changing amelogenin self-assembly and molecular structure in the presence of mineral, one would expect that the enamel matrix appearance varies according to location and at interfaces with crystallites. To refine our models and concepts of enamel formation requires the analysis of mineralizing enamel *in situ* and the application of high-resolution imaging techniques that provide nanometer resolution. The data presented here demonstrate that HIM imaging, especially in combination with FE-SEM, is a very promising approach to achieve this goal. Taking advantage of the much smaller wavelength of helium ions compared to electrons and a small convergence angle of the helium ions on the sample, as explained briefly above in Methods (Figure 3), an outstanding depth of field is achieved in HIM imaging as seen in Figures 4–6. These images also illustrate quite well the advantage of charge neutralization available in HIM imaging through the use of a flood gun. Although all of the analyzed samples are uncoated biological samples embedded in acrylic resin and thus completely insulating material, there is no charge build up in HIM imaging, which is on the contrary notorious in electron microscopy of this sample type. Especially the very thin crystallites that protrude from the sample in various directions, seen in mature enamel depicted in Figure 3 and early maturation stage enamel in Figure 4, can be imaged at very high resolution in HIM without developing charges and appear equally crisp in all planes. The problem of charges developing in electron microscopy can be addressed using imaging under variable pressure conditions albeit at the significant expense of analytical resolution. Although it seems apparent in these figures that the needle shaped morphology is indicative of enamel crystallites, the unequivocal distinction between protein and mineral, and ideally mineral phase identification, requires additional sample analysis. Conventionally, mineral phase identification is performed using selected area diffraction analyses in (TEM) of ultra thin sections. However, using an electron backscattered diffraction system (ESBD) offers an alternative approach for mineral phase identification on en bloc samples with polished surface. Unfortunately, this kind of detector was not available on the microscopes used in this study.

The much greater depth of field in HIM imaging combined with helium ion beam-sample interactions that remain very much on the sample surface, on the one hand, and the deeper reaching electron beam-sample interactions in FE-SEM (Figure 3), on the other hand, explain the different imaging results illustrated in Figure 7. Using FE-SEM, structural details and organization of the organic material are revealed in the same stage of enamel development, but in equally prepared samples (Figures 7A,B) these features cannot be discerned using HIM (Figures 7C,D).

For this study we have used immunohistochemistry in conjunction with high-resolution microscopy to only identify amelogenin (Figures 8A–C, 9) and explore the usefulness of HIM in comparison to FE-SEM for the detection of immuno-gold labels. However, it is known that ameloblastin and enamelin are also present at the mineralization front (Gallon et al., 2013; Mazumder et al., 2014) and required for enamel formation (Fukumoto et al., 2004; Hu et al., 2008; Bartlett, 2013). It is therefore important to keep in mind that the presented data do not preclude the presence of these matrix proteins in newly secreted matrix and enamel prisms formation.

The data presented here demonstrate that HIM imaging, especially in combination with FE-SEM, is a very promising approach to further our understanding of enamel matrix organization and enamel mineralization *in situ*. The use of these two high-resolution imaging techniques together on a given sample provides complementary information on the structural three dimensional organization of the mineralizing enamel matrix and its relation to crystallite formation.

### Conflict of interest statement

The authors declare that the research was conducted in the absence of any commercial or financial relationships that could be construed as a potential conflict of interest.
